# Removal of reactive blue 19 from aqueous solution by pomegranate residual-based activated carbon: optimization by response surface methodology

**DOI:** 10.1186/2052-336X-12-65

**Published:** 2014-03-28

**Authors:** Elham Radaei, Mohammad Reza Alavi Moghaddam, Mokhtar Arami

**Affiliations:** 1 Department of Civil and Environmental Engineering, Amirkabir University of Technology (AUT), Tehran, Iran; 2 Department of Textile Engineering, Amirkabir University of Technology (AUT), Tehran, Iran

**Keywords:** Reactive dye, Adsorption, Pomegranate residual, Response surface methodology

## Abstract

**Background:**

In this research, response surface methodology (RSM) was applied to optimize Reactive Blue 19 removal by activated carbon from pomegranate residual. A 2^4^ full factorial central composite design (CCD) was applied to evaluate the effects of initial pH, adsorbent dose, initial dye concentration, and contact time on the dye removal efficiency.

**Methodology:**

The activated carbon prepared by 50 wt.% phosphoric acid activation under air condition at 500°C. The range of pH and initial dye concentration were selected in a way that considered a wide range of those variables. Furthermore, the range of contact time and adsorbent dose were determined based on initial tests. Levels of selected variables and 31 experiments were determined. MiniTab (version 16.1) was used for the regression and graphical analyses of the data obtained.

**Results:**

It was found that the decrease of initial dye concentration and the increase of initial pH, adsorbent dose, and contact time are beneficial for improving the dye removal efficiency. Analysis of variance (ANOVA) results presented high R^2^ value of 99.17% for Reactive Blue 19 dye removal, which indicates the accuracy of the polynomial model is acceptable.

**Conclusions:**

Initial pH of 11, adsorbent dose of 1.025 g/L, initial dye concentration of 100 mg/L, and contact time of 6.8 minutes found to be the optimum conditions. Dye removal efficiency of 98.7% was observed experimentally at optimum point which confirmed close to model predicted (98.1%) result.

## Introduction

Many industries, especially textile and food industries often use dyes and pigments to color their products. As a result, these industries often discharge large amounts of colored effluents due to unfixed dyes on fibres or food during coloring and washing steps [1]. Due to the disposal of these effluents into the receiving water body may cause severe damage to aquatic biota and humans due to mutagenic and carcinogenic effects [2-4], it is of great importance to provide waste-treatment facilities for minimizing these substances in the effluents before discharge.

There are several methods available for treatment of dye-containing wastewater, such as membrane [5], electrochemical [6], coagulation/flocculation [7], and biological [8]. The adsorption technique has been found not only to be effective, but also practical in application for the dye-containing wastewater treatment, due to its high efficiency, simplicity, ease of operation, and the availability of a wide range of adsorbents [9,10]. Activated carbons (ACs) are widely used as the most efficient adsorbents. The chemical activation is often used to produce ACs and it involves mixing the feedstock with a chemical activating agent such as H_3_PO_4_, ZnCl_2_, and KOH [11-13].

Conventional and classical methods of studying a process by maintaining other factors involved at an unspecified constant level does not depict the combined effect of all the factors involved. Response Surface Methodology (RSM) is a collection of mathematical and statistical techniques useful for developing, improving, and optimizing processes and can be used to evaluate the relative significance of several affecting factors even in the presence of complex interactions. The main objective of RSM is to determine the optimum operational conditions for the system or to determine a region that satisfies the operating specifications [14]. Many research groups applied this method for removal of different pollutants by adsorption process [14-19].

The aim of the present study is to optimize and model the removal of Reactive Blue 19 from aqueous solution by activated carbon derived from pomegranate residual using RSM. The relationship between dye removal efficiency and four main independent parameters including initial pH, initial dye concentration, adsorbent dose, and contact time were evaluated by applying central composite design (CCD).

## Methods

### Materials and activated carbon preparation

Dye solution was prepared by dissolving Reactive blue 19 (RB19) which was provided by the Alvan Sabet Company and is widely used in textile industries in Iran. The chemical structure and characteristics of the selected dye is presented in Table 1. The solution pH measurement was carried out using a 340i/SET pH meter (WTW-Germany) and was adjusted by 1 M hydrochloric acid or 1 M sodium hydroxide. The dye solution and adsorbent was agitated by a jar test at 150 rpm agitation speed at ambient temperature 25°C. A six beaker jar test apparatus from Zag-Chemi Company in Iran was used to simulate the adsorption process. All samples were filtered through glass fibre filters GF/A. The clear supernatants were analyzed for RB19 dye concentrations using a UV–vis HACH spectrophotometer (DR/4000).

**Table 1:** Chemical structure and characteristics of RB19

**Characteristics**	**Values**
Molecular formula	C_22_H_16_N_2_Na_2_O_11_S_3_
λmax (nm)	594
Molecular weight (MW)	626.54
Chemical structure	

In this study, pomegranate residual was collected from Meykhosh juice industry in Yazd/Iran. Pomegranate residual has been dried in an oven for 2 h at 100°C until a constant weight was reached. It was then ground in a ball mill and passed through sieve No.8. They were soaked for 24 h in the ratio of 1:1 (w/v) with 50 wt.% phosphoric acid at room temperature. The sample is then decanted, dried in a muffle furnace for 1 h at 500°C. Then the samples were washed sequentially several times with hot distilled water, until pH of the washing solution became neutral. In the last step, activated carbon (AC) was powder and sieved by the No. 200 mesh.

**Figure 1:**
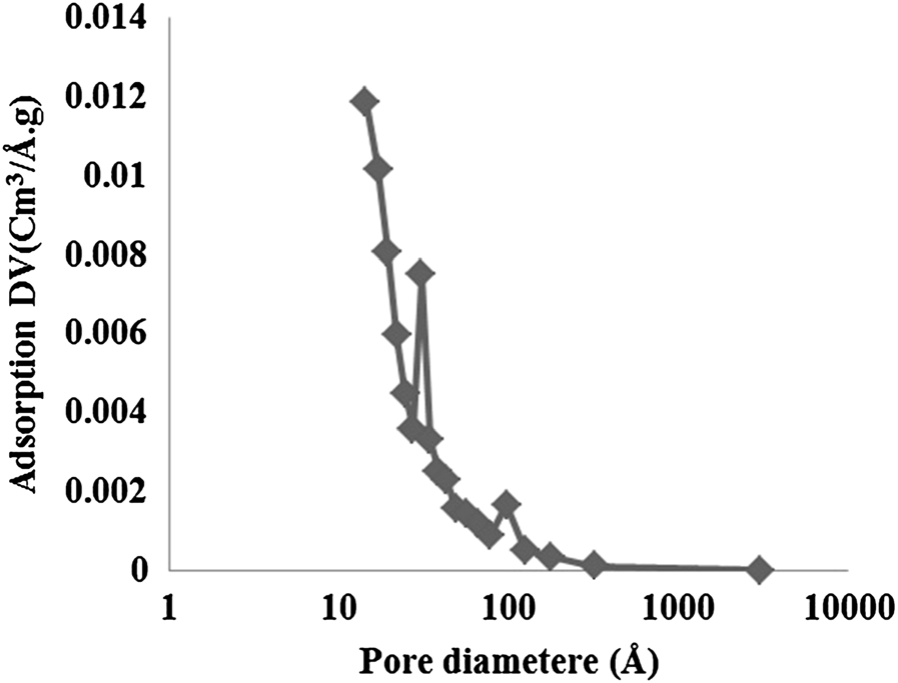
Pore size distribution of AC.

The textural properties of adsorbent were also tested by N_2_ adsorption/desorption isotherms at 77 K using an Autosorb 1 analyzer (Quantachrome Corporation, USA). The specific surface area (S_BET_) was calculated by Brun- auer–Emmett–Teller (BET) method. The textural characteristics of AC are shown in Table 2. The pore size distribution was determined by using the Barrett–Joyner– Halenda (BJH) method (Figure 1).

**Table 2:** Textural properties obtained by N2 adsorption/desorption studies

**Parameters**		**Values**
BET surface area (m^2^/g)	BET^a^	825.46
Pore volume (cm^3^/g)	BJH adsorption^b^	0.3455
Pore diameter (Å)	BJH adsorption	14.35

^a^Computed in the P/P_0_ range 0.05–0.30.

^b^BJH (Barrett Joyner Halenda) cumulative adsorption pore volume for pores in range of 0 and 5000 Å diameter.

### Experimental design

A central composite design (CCD) was employed for determining the optimum condition for the dye removal. A total of 31 experiments were carried out according to a 2^4^ full factorial CCD, consisting of 16 factorial experiments (coded to the usual ± 1 notation), 8 axial experiments (on the axis at a distance of ± α from the center), and 7 replicates (at the center of the experimental domain).

The value of α for rotatability depends on the number of points in the factorial portion of the design, which is given in equation (1):

(1) $$\alpha ={\left(N_{F}\right)}^{1/4\;}$$

where N_F_ is the number of points in the cube portion of the design (N_F_ =2^k^, k is the number of factors). Therefore, α is equal to (2^4^)^1/4^ = 2 according to equation (1).

**Figure 2:**
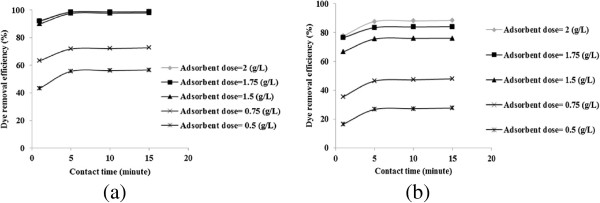
Results of initial tests for determining of adsorbent dose and contact time, initial pH = 11: (a) initial dye concentration = 300 mg/L and (b) initial dye concentration = 500 mg/L.

The range of pH and initial dye concentration were selected in a way that considered a wide range of those variables. The dye concentration was selected between 100 to 500 mg/L. This range is based on determined actual concentration of textile wastewaters in Iran. The range of contact time and adsorbent dose were determined based on initial tests. The results of initial tests are shown in Figure 2. As demonstrated in the figures, the dye was rapidly adsorbed at a high rate in the first 5 minute, and after 10 minutes leveled off. Therefore, the range of contact time was considered between 1 minute and 10 minutes. Due to a slight difference between dye removal efficiencies for adsorbent dose of 2 g/L and 1.75 g/L, the maximum amount of adsorbent dose was considered 1.75 g/L. In addition, adsorbent dose of lower than 0.75 g/L was not enough efficient to remove RB19.

Levels of selected variables are presented in Table 3. For statistical calculations, the variables X_i_ (the real value of an independent variable) were coded as x_i_ (dimensionless value of an independent variable) according to equation (2):

(2) $$x_{i}=\left(X_{i}–X_{0}\right)/\mathrm{\Delta X}\;$$

where X_0_ is the value of X_i_ at the center point and ΔX represents the step change.

**Table 3:** Experimental range and levels of the independent variables

**Parameters**		**Levels**				
		**-α**	**-1**	**0**	**1**	**α**
Initial pH	x_1_	3	5	7	9	11
Adsorbent dose (g/L)	x_2_	0.75	1.00	1.25	1.50	1.75
Initial dye concentration (mg/L)	x_3_	100	200	300	400	500
Contact time (min)	x_4_	1	3	5	7	9

The behavior of the adsorption process is explained by the following empirical second-order polynomial model equation (3):

(3) $$Y_{i}=b_{0}+\sum_{i=1}^{n}b_{i}x_{i}+\sum_{i=1}^{n}b_{\mathrm{ii}}{x_{i}}^{2}+\sum_{i=1}^{n‒1}\sum_{j=i+1}^{n}b_{\mathrm{ij}}x_{i}x_{j}$$

where Y is the predicted response (dye removal efficiency), b_0_ the constant coefficient, b_i_ the linear coefficients, b_ii_ the quadratic coefficients, b_ij_ the interaction coefficients and x_i_, x_j_ are the coded values of the variables. MiniTab (version 16.1) was used for the regression and graphical analyses of the data obtained. The reliability of the fitted model was justified through ANOVA and the coefficient of R^2^.

## Results

### Statistical analysis

The experimental design matrix, the experimental results and the predicted dye removal efficiency are presented in Table 4. The final model is expressed by equation (4):

(4) $$\begin{array}{ll} Y= & 69.3371+2.3943\;x_{1}+9.0655\;x_{2}‒10.7734x_{3}\\ & +1.2223\;x_{4}+3{{.5562x}_{1}}^{2}‒1{{.6346x}_{2}}^{2}\\ & +0{{.4977\;x}_{3}}^{2}‒0{{.6212x}_{4}}^{2}+0.8284x_{1}x_{2}\\ & +0.8070\;x_{1}x_{3}\\ & +0.0549\;x_{1}x_{4}‒0.0465x_{2}x_{3}‒0.0007x_{2}x_{4}\\ & +0.1210\;x_{3}x_{4} \end{array}$$

**Table 4:** RSM design and its observed and predicted values

**Run**	**Initial pH (x**_**1**_**)**	**Adsorbent dose (x**_**2**_**)**	**Initial dye concentration (x**_**3**_**)**	**Contact time (x**_**4**_**)**	**Dye removal (%)**	
					**Experimental**	**Predicted**
1	5	1	200	3	70.46	70.99
2	9	1	200	3	71.57	72.39
3	5	1.5	200	3	89.35	87.55
4	9	1.5	200	3	91.64	92.28
5	5	1	400	3	49.39	47.68
6	9	1	400	3	51.40	52.31
7	5	1.5	400	3	64.34	64.06
8	9	1.5	400	3	72.00	72.01
9	5	1	200	7	73.06	73.08
10	9	1	200	7	74.41	74.71
11	5	1.5	200	7	90.54	89.65
12	9	1.5	200	7	92.84	94.59
13	5	1	400	7	50.87	50.25
14	9	1	400	7	53.28	55.11
15	5	1.5	400	7	67.43	66.63
16	9	1.5	400	7	75.32	74.80
17	3	1.25	300	5	75.98	78.77
18	11	1.25	300	5	91.19	88.35
19	7	0.75	300	5	45.68	44.66
20	7	1.75	300	5	79.95	80.92
21	7	1.25	100	5	93.52	92.87
22	7	1.25	500	5	49.17	49.78
23	7	1.25	300	1	63.94	64.40
24	7	1.25	300	9	69.80	69.29
25	7	1.25	300	5	69.27	69.33
26	7	1.25	300	5	70.50	69.33
27	7	1.25	300	5	69.27	69.33
28	7	1.25	300	5	68.30	69.33
29	7	1.25	300	5	71.27	69.33
30	7	1.25	300	5	67.47	69.33
31	7	1.25	300	5	69.27	69.33

**Figure 3:**
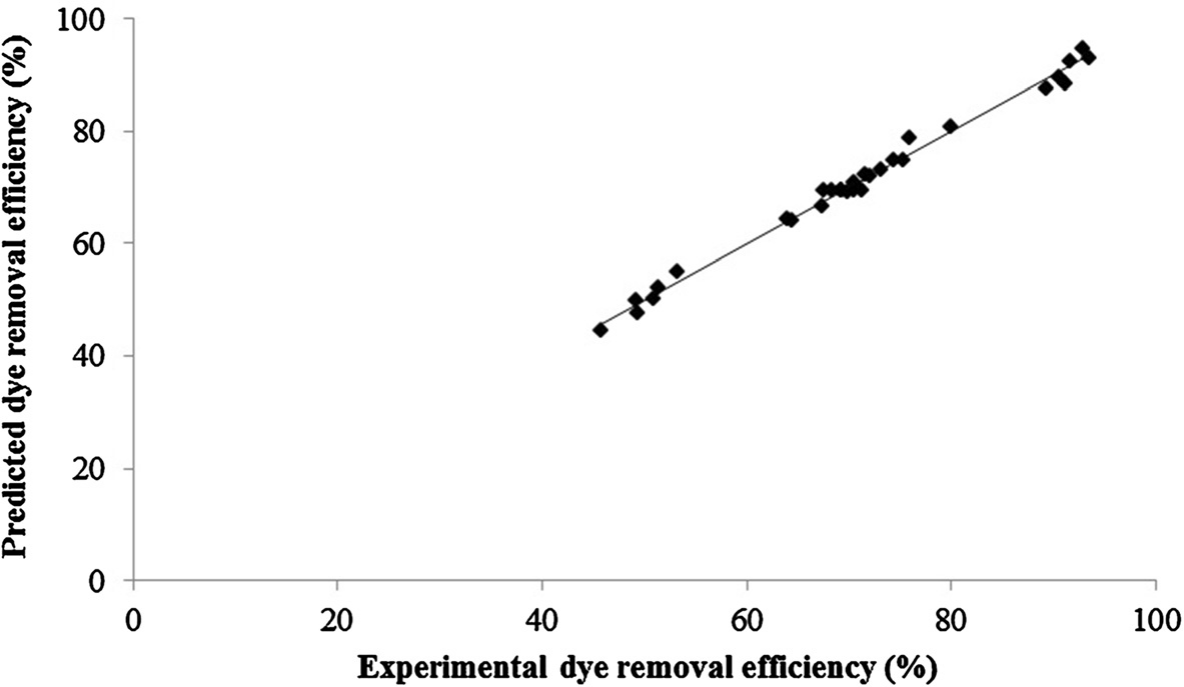
Parity plot for the experimental and predicted value of RB19 removal efficiency (%).

Statistical regression coefficients for RB19 dye removal efficiency (%) is provided in Table 5. Amounts of P (P < 0.05) for all independent parameters confirms that four selected factors are significant. However, it was found that all square and interaction terms except x_1_^2^ and x_2_^2^ (P values of 0.000) were insignificant to the response. ANOVA for the selected dye removal is also listed in Table 6. In this case, the P-value of 0.000 (P < 0.05) for regression model equation implies that the second-order polynomial model fitted to the experimental results well. The lack-of-fit was also calculated from the experimental error (pure error) and residuals. “F-value of Lack-of-fit” of 2.24 implies the significance of model correlation between the variables and process response for dye removal. Additionally, the value of R^2^ = 99.17% and R^2^ (adj) =98.44% confirm the accuracy of the model. Furthermore, parity plot for the experimental and predicted value of RB19 removal efficiency (%) is demonstrated in Figure 3.

**Table 5:** Statistical regression coefficients for RB19 removal efficiency (%) in coded units

**Term**	**Coefficient**	**SE Coefficient**	**T**	**P**
Constant	69.3371	0.6393	108.465	0.000
x_1_	2.3943	0.3452	6.935	0.000
x_2_	9.0655	0.3452	26.259	0.000
x_3_	−10.7734	0.3452	−31.206	0.000
x_4_	1.2223	0.3452	3.541	0.003
x_1_^2^	3.5562	0.3163	11.244	0.000
x_2_^2^	−1.6346	0.3163	−5.168	0.000
x_3_^2^	0.4977	0.3163	1.573	0.135
x_4_^2^	−0.6212	0.3163	−1.964	0.067
x_1_x_2_	0.8284	0.4228	1.959	0.068
x_1_x_3_	0.8070	0.4228	1.909	0.074
x_1_x_4_	0.0549	0.4228	0.130	0.898
x_2_x_3_	−0.0465	0.4228	−0.110	0.914
x_2_x_4_	−0.0007	0.4228	−0.002	0.999
x_3_x_4_	0.1210	0.4228	0.286	0.778

**Table 6:** ANOVA for RB19 removal efficiency (%)

**Source**	**DF**	**Seq SS**	**Adj SS**	**Adj MS**	**F**	**P**
Regression	14	5455.27	5455.27	389.66	136.22	0.000
Linear	4	4931.44	4931.44	1232.86	430.99	0.000
Square	4	502.12	502.12	125.53	43.88	0.000
Interaction	6	21.72	21.72	3.62	1.27	0.327
Residual error	16	45.77	45.77	2.86		
Lack-of-fit	10	36.11	36.11	3.61	2.24	0.168
Pure error	6	9.66	9.66	1.61		
Total	30	5501.04				

Note: R^2^ = 99.17%, R^2^ (adj) = 98.44%.

**Figure 4:**
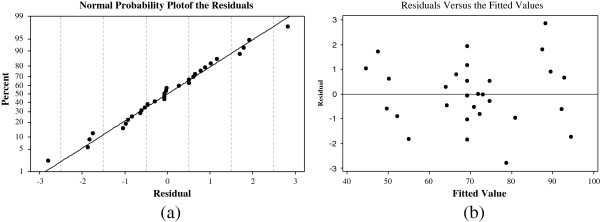
(a) Normal probability plot and (b) residual versus fit plot for RB19 removal efficiency (%).

In addition, normal probability and residuals versus fitted values plots for RB19 removal efficiency are illustrated in Figure 4. Normal probability plot is a suitable graphical method for judging the normality of the residuals [18]. As seen in Figure 4(a), the normality assumption was relatively satisfied as the points in the plot form a fairly straight line. The reliability of the model was also examined with the plot of residuals versus fits in Figure 4(b). As Figure 4(b) shows, the number of increasing or decreasing points was significantly close; patterns of increasing residuals and increasing fits were similar; and, positive and negative residuals were scattered in same range. As a result, Figure 4 shows that the model is adequate to describe RB19 removal efficiency by response surface methodology for AC.

### Response surface and counter plotting for evaluation of operational parameters

**Figure 5:**
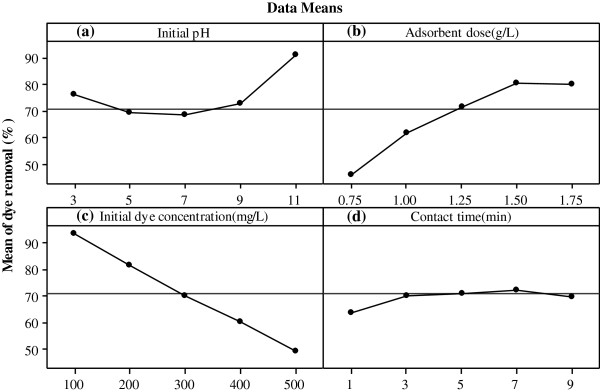
Main effect plots of parameters for RB19 removal efficiency: (a) initial pH, (b) adsorbent dose, (c) initial dye concentration and (d) contact time.

The main effect of each parameter on the dye removal efficiency is showed in Figure 5. As illustrated in Figure 5(a, b, c), by increasing of initial pH and adsorbent dose, and decreasing of initial dye concentration, the dye removal efficiency improved. Moreover, as shown in Figure 5(d), adsorption efficiency rapidly increased with an increase in contact time within the first minutes. Then, the rate of adsorption was found to be relatively slow and then constant. As shown in this Figure, the dye removal is highly dependent on adsorbent dose and initial dye concentration, and the initial pH and contact time slightly influenced the process efficiency.

**Figure 6:**
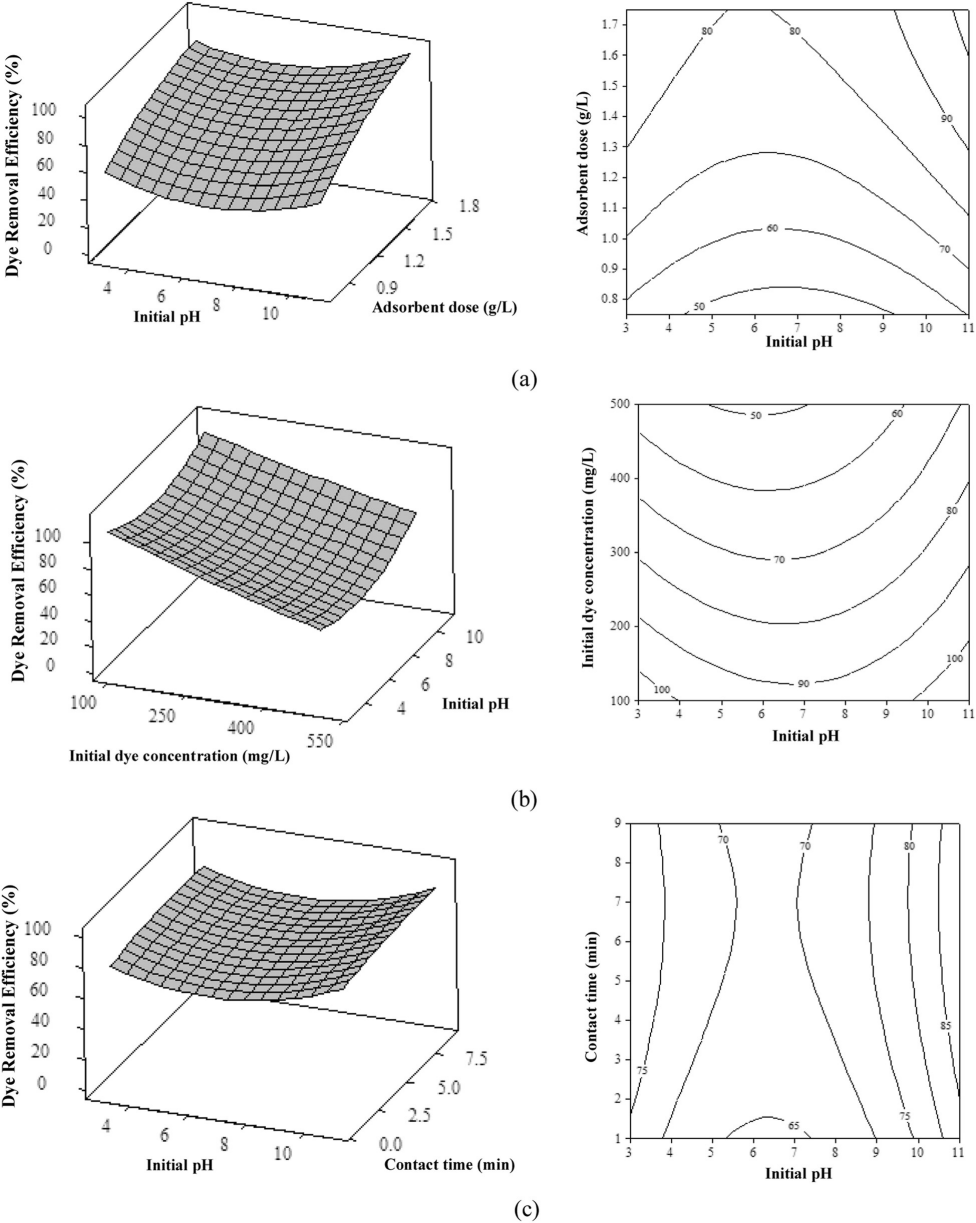
Surface corresponding contour plot as a function of initial pH and: (a) adsorbent dose (initial dye concentration of 300 mg/l and contact time of 5 min) (b) initial dye concentration (adsorbent dose of 1.25 g/L and contact time of 5 min) (c) contact time (initial dye concentration of 300 mg/l and adsorbent dose of 1.25 g/L).

For a better explanation of the independent variables and their interactive effects on the dye removal, 3D plots and its corresponding contour plots are represented in Figure 6. At constant value of the initial dye concentration (300 mg/L), when the adsorbent dose and initial pH increase, the dye removal efficiency increases and finally reaches to 100% (Figure 6(a)). Moreover, for given initial dye concentration of 100 mg/L and adsorbent dose of 1.25 g/L, dye removal efficiency is almost independent of initial pH (Figure 6(b)). This independency is due to the existence of numerous active sites for the adsorption, whereas the independency was decreased with increasing of initial dye concentration. As shown in Figure 6(c), there are no significant changes on the dye removal surface with the increase of contact time and initial pH.

### Process optimization

In order to determine the optimum conditions by the adsorption process, the desired aim in terms of RB19 removal efficiency was defined as target to achieve 98% removal efficiency. The optimum values of the process parameters were calculated in coded units (x_i_) and then converted into uncoded units (X_i_) using Equation ([1]). Initial pH of 11, adsorbent dose of 1.025 g/L, initial dye concentration of 100 mg/L and contact time of 6.8 minutes found to be the optimum conditions by the model. The optimum condition was repeated three times and dye removal efficiencies of 98.4, 98.6, and 99.1% were resulted. The average of 98.7% dye removal efficiency was found close to the model prediction of 98.1%.

## Discussion

According to the results, contact time is the least important parameter, which is reported by other research groups [20,21]. Furthermore, by increasing of initial pH and adsorbent dose, and decreasing of initial dye concentration, the dye removal efficiency improved. These results are in good agreement with of previous studies [22,23].

The effect of the four selected independent parameters and interactions among the RSM were analyzed which was shown that some interactions like (x_1_^2^ and x_2_^2^) influenced the adsorption performance as well as all selected parameters. ANOVA showed a high R^2^ value of regressions model equation (R^2^ = 0.9917), thus ensuring a satisfactory adjustment of the second-order regression model with the experimental data. The optimum RB19 removal efficiency were found at initial pH of 11, adsorbent dose of 1.025 g/L, initial dye concentration of 100 mg/l and contact time of 6.8 min. An experiment was performed in optimum conditions which confirmed that the model and experimental results are in close agreement (98.7% compared to 98.1% for the model).

## Conclusions

In this research, response surface methodology was applied as an experimental design to explore the optimal conditions for RB19 dye removal from aqueous solutions by activated carbon prepared by pomegranate residual. The effect of four operating variables of adsorption process including initial pH, adsorbent dose, initial dye concentration, and contact time were examined. The BET method showed that the average S_BET_ of AC was 825.46 m^2^ g^−1^. The results of this investigation presented that RSM is a powerful statistical optimization and modeling tool for RB19 removal using adsorption process.

## Competing interests

The authors declare that they have no competing interests.

## Authors’ contributions

This work is part of the Master thesis of ER where MRA, Supervisor of the thesis, suggested the problem and read the paper and MR, Co-Supervisor of the thesis, helped in analysis of experiments. All authors read and approved the final manuscript.
